# Standard: human intestine-on-a-chip

**DOI:** 10.1186/s13619-024-00198-7

**Published:** 2024-08-05

**Authors:** Haitao Liu, Yaqing Wang, Xu Zhang, Min Zhang, Peng Wang, Jing Shang, Zhongqiang Li, Likun Gong, Jiabin Guo, Wei Sun, Jingbo Pi, Xianliang Li, Wei Ding, Dianbing Wang, Zhongyu Li, Jingzhong Zhang, Lan Wang, Xingchao Geng, Ruifu Yang, Pingkun Zhou, Wanjin Tang, Xian’en Zhang, Chunying Chen, Shengli Yang, Jianhua Qin

**Affiliations:** 1 Division of Biotechnology, Dalian Institute of Chemical Physics, Chinese Academy of Sciences, Dalian, China; 2 University of Science and Technology of China, Hefei, China; 3 Suzhou Institute for Advanced Research, University of Science and Technology of China, Suzhou, China; 4 China Pharmaceutical University, Nanjing, China; 5 China National Institute of Standardization, Beijing, China; 6 Shanghai Institute of Materia Medica, Chinese Academy of Sciences, Shanghai, China; 7 Chinese People’s Liberation Army (PLA) Center for Disease Control and Prevention, Beijing, China; 8 Tsinghua University, Beijing, China; 9 China Medical University, Shenyang, China; 10 Department of HBP Surgery, Beijing Chao Yang Hospital, the Capital Medical University, Beijing, China; 11 SPH KDL Health Beijing, Shanghai Pharma, Beijing, China; 12 Institute of Biophysics, Chinese Academy of Sciences, Beijing, China; 13 Dalian Minzu University, Dalian, China; 14 Suzhou Institute of Biomedical Engineering and Technology, Chinese Academy of Sciences, Suzhou, China; 15 Dalian Xin’en Medical Technology Co., LTD, Dalian, China; 16 National Institutes for Food and Drug Control, Beijing, China; 17 Institute of Microbiology and Epidemiology, Academy of Military Medical Sciences, Beijing, China; 18 Institute of Radiation Medicine, Chinese Academy of Medical Sciences, Tianjin, China; 19 Shenzhen University of Advanced Technology, Shenzhen, China; 20 National Center for Nanoscience and Technology, Beijing, China

**Keywords:** Organs-on-chips, Intestine-on-a-chip, Microphysiological system, Standard

## Abstract

Organs-on-chips are microphysiological systems that allow to replicate the key functions of human organs and accelerate the innovation in life sciences including disease modeling, drug development, and precision medicine. However, due to the lack of standards in their definition, structural design, cell source, model construction, and functional validation, a wide range of translational application of organs-on-chips remains a challenging. “Organs-on-chips: Intestine” is the first group standard on human intestine-on-a-chip in China, jointly agreed and released by the experts from the Chinese Society of Biotechnology on 29th April 2024. This standard specifies the scope, terminology, definitions, technical requirements, detection methods, and quality control in building the human intestinal model on a chip. The publication of this group standard will guide the institutional establishment, acceptance and execution of proper practical protocols and accelerate the international standardization of intestine-on-a-chip for translational applications.

## Scope

This document specifies the ethical requirements, Technical requirements, and detection methods for construction and evaluation of human intestine-on-a-chip.

This document is applicable to the construction, Detection and evaluation activities of functional intestine-on-a-chip

## Normative references

The contents of the following documents are essential provisions of this document through normative reference. For referenced documents with specified dates, only the version corresponding to that date is applicable to this document; for documents without specified dates, the latest version (including all amendments) is applicable to this document:

- GB/T 16886.5–2017 Biological evaluation of medical devices-Part 5: Tests for in vitro cytotoxicity
- GB/T 38736–2020 Ethical Requirements of Human Biobanking
- GB/T 42466–2023 Technical Specification for Pluripotent Stem Cells Management of Biobanking
- WS 213 Diagnostic for Hepatitis C
- WS 293 Diagnostic Criteria for HIV/AIDS
- WS 299 Diagnostic Criteria for Viral Hepatitis B
- T/CSB 0003–2024 General Terminology of Organs-on-chips
- T/CSCB 0001–2020 General Requirements for Stem Cells
- T/CSCB 0013–2022 Human Intestinal Organoid

## Terms and definitions

The terms defined in T/CSB 0003–2024 and the following terms and definitions apply to this document:

### Intestine-on-a-chip

An intestinal microphysiological system that can reflect the key structures and biofunctions (such as absorption, secretion, metabolism, and barrier properties) of human intestine by seeding multiple cells (e.g., intestinal epithelial cells and vascular endothelial cells) and reconstructing microenvironmental elements (e.g., tissue-tissue interface and fluid flow), or applying cyclic tension to mimic intestinal peristalsis (T/CSB 0003–2024, 5.5).

### Intestinal barrier

The integration of structures and functions that ensure the efficient absorption of nutrients and barricade harmful substances (e.g., bacteria and toxins) from permeating through the intestinal mucosa into the body's organs and bloodstream (Chinese Terms in Parenteral and Enteral Nutrition-2019).

### Intestinal villus

Tiny projections on the surface of the mucous membrane of the small intestine, of which a single layer of columnar epithelium is on the surface and the mucosal lamina propria is in the axis (Chinese Terms in Histology and Embryology (2nd Edition)-2014).

### Intestinal microenvironment

The summation of biophysical and biochemical factors in intestinal tissue that affect cell growth and functions, including various cell types, extracellular matrix, fluid stimuli, physicochemical gradients and intestinal flora (Kim 2012; Guo 2018).

### Intestinal epithelial cells

Single-layered cells that make up the luminal surface (inner layer) of the gastrointestinal tract, including the small intestine and colon. Intestinal epithelial cells of the small intestine include Paneth cells and undifferentiated cells (Shin 2018).

### Organoid

Self-assembled 3D microtissues derived from stem cells or organ-specific precursor cells through self-renewal, differentiation, and self-organization, which consist of multiple cell types and can partially reflect the key structures and specific functions of their source tissue or organ (T/CSB 0003–2024, 3.3).

### Intestinal organoid

Self-assembled intestinal microtissues derived from stem cells through self-renewal, differentiation, and self-organization, which consist of multiple types of mature intestinal epithelial cells and can partially reflect the key structures and tissue-specific functions of in vivo intestinal epithelium.

### Intestinal barrier permeability

The ability of compounds to pass through the intestinal barrier. Intestinal barrier permeability indirectly impacts the absorption of compounds in the body, focusing on the fraction of the absorbed dose rather than systemic bioavailability. It is directly linked to the rate at which compounds transfer across the intestinal barrier (Kwon 2021).

### Bioavailability

The relative amount and rate at which a bioactive substance (such as drugs, nutrients, or toxins) is absorbed into the body’s circulation, depending on various factors, including intestinal barrier permeability, the solubility of the substance, its molecular size, and the presence of transporters. Bioavailability reflects the information related to the time and dose at which substances enter the bloodstream, tissue organ uptake, and pharmacological effects (Yang and Chen 2018).

### Transepithelial electrical resistance, TEER

The resistance to ion flow across cell layers. TEER is related to the integrity of tight junctions between cells and can reflect the integrity and permeability of the cellular barrier (Odijk 2015; van der Helm 2016; Guo 2024).

### Apparent permeability coefficient, Papp

The relative rate at which compounds pass through the intestinal barrier per unit area of the intestine over a certain period. Papp is an important indicator for assessing the absorption of compounds in the intestine.

The formula for calculating the apparent permeability coefficient is

Papp = (dQ/dt) / (A × c_0_),

where:

- (dQ/dt) is the amount of compound passing through the intestinal barrier per unit time,
- A is the surface area of the intestine,
- c_0_ is the initial concentration of the compound.

## Ethical requirements

### General principles

When the preparation and evaluation process of an intestine-on-a-chip involves the use of human primary tissues or cells, which raises ethical concerns, ethical approval should be obtained, and informed consent should be obtained to ensure the privacy protection of the donors.

### Informed consent

Prior to the collection of human samples, written informed consent should be obtained from the donors, clearly outlining the rights and responsibilities of both the donors and the sample collection entity. Informed consent should comply with the requirements of Sect. 5.3 of GB/T 38736–2020.

### Ethical review

The establishment and research protocols of the intestinal chip should be reviewed and approved by the ethics review committee of the project's sponsoring and primary executing institution.

### Privacy protection

Human sample donors have the right to personal privacy, and the confidentiality and protection of their personal privacy information should comply with the requirements of Chapter 6 of GB/T 38736–2020.

## Construction of intestine-on-a-chip

### Technical requirements

#### Fabrication of the chip

##### Design of the chip

The structure of the intestinal chip carrier typically includes two chambers separated by a porous membrane: one chamber for seeding intestinal epithelial cells and the other chamber for seeding vascular endothelial cells, with fluid environments present in both chambers (Li 2017).

Note 1: The structure of the intestinal chip carrier can also take other reasonable forms, such as using extracellular matrix instead of a porous membrane.

Note 2: Depending on the experimental purposes, other cells such as immune cells may also be added to the chambers on both sides of the porous membrane.

##### Material selection for the chip

The commonly used materials for the intestine-on-a-chip should preferably be PDMS.

Other materials for the intestine-on-a-chip typically include PMMA, PS, and PC.

The material of the porous membrane can be selected from PDMS, PC, PET, and nylon, with pore sizes ranging from 0.01 μm to 10 μm.

##### Methods for chip fabrication

PDMS chips are commonly fabricated using soft lithography method.

Chips made of materials such as PMMA, PS, and PC are preferably manufactured using techniques such as injection molding, machining and 3D printing.

For PDMS chips, plasma bonding is typically used for processing; for plastic materials such as PMMA, PS, and PC, methods such as heat sealing, ultrasonic sealing, and adhesive sealing are commonly employed.

##### Quality control for chip

The cytotoxicity of chips should be tested using methods outlined in GB/T 16886.5–2017. The chips should meet the non-cytotoxicity requirements outlined in Chapter 8 of GB/T 16886.5–2017.

#### Seeding cells in the chip

##### Cell components

Intestine-on-a-chip should contain human intestinal epithelial cells (Guo 2021).

Note: Intestinal epithelial cells typically include Caco-2 cell line, primary human intestinal cells, or intestinal organoids, among others.

When simulating the structure and function of the intestinal epithelial-endothelial barrier, intestinal chips should include endothelial cells.

Note: Endothelial cells should preferably be derived from human umbilical vein endothelial cells, human intestinal microvascular endothelial cells, or endothelial cells differentiated from stem cells (such as induced pluripotent stem cells and embryonic stem cells), with a maximum passage number of 10.

When simulating intestinal immune function, immune cells should be added to the endothelial cell side of the intestinal chip.

Note: Immune cells typically include monocytes and macrophages, among others.

##### Requirements of cell performance

The typical requirements for cell performance in the chip include:

a) Cell Morphology

The morphology of various cell types before seeding should correspond to their normal morphology, which can be referenced on the ATCC website. For example:

- Caco-2 cells exhibit cuboidal, epithelial-like, or giant-cell-like morphology, with some cells containing large vacuoles.
- HT-29-MTX cells are round or square-shaped and tend to grow in clusters.
- Intestinal organoids appear as spheroids or bud-like structures with a central lumen, surrounded by tightly packed columnar epithelial cells.
- Endothelial cells have flat polygonal or spindle-shaped morphology.
- In immune cells, monocytes often have kidney-shaped or horseshoe-shaped nuclei, with varying cell shapes including round and polygonal; macrophages are round or elliptical, with short projections, and activated ones may extend irregularly-shaped pseudopods.

b) Cell Chromosome Karyotype

The normal chromosome karyotype of cells should be 46, XY or 46, XX.

c) Microbiological Testing

Microbiological testing indicators include:

i) Bacteria and Fungi

Testing should follow the "Sterility Test" method outlined in the "Pharmacopoeia of the People's Republic of China (2020 Edition)." After culturing by membrane filtration or direct inoculation for no less than 14 days, observe whether the culture medium becomes turbid, or take culture fluid smears, stain, and microscopically examine.

ii) Chlamydia

Testing should follow the "Chlamydia Test" method outlined in the "Pharmacopoeia of the People's Republic of China (2020 Edition)." Testing can be done using Chlamydia culture method or indicator cell culture method (DNA staining), or other methods approved by the national drug regulatory authority.

iii) HIV

Testing should be conducted using the WS 293 nucleic acid method, with HIV-related RNA below the detection limit in routine PCR.

iv) HBV

Testing should be conducted using the WS 299 nucleic acid method, with HBV-related RNA below the detection limit in routine PCR.

v) HCV

Testing should be conducted using the WS 213 nucleic acid method, with HCV-related RNA below the detection limit in routine PCR.

vi) Exogenous Viral Factors

Testing should follow the "Exogenous Viral Factor Test" method outlined in the "Pharmacopoeia of the People's Republic of China (2020 Edition)," using methods such as cell culture, inoculation of suckling mice or chicken embryos, nucleic acid amplification technology, etc.

When human stem cells are used in the chip, they should meet the requirements of Chapter 5 and Chapter 6 of T/CSCB 0001–2020, as well as Chapter 8 of GB/T 42466–2023.

When intestinal organoids are used in the chip, the cellular composition and proportions of the organoids should comply with the requirements of Sect. 6.4 of T/CSCB 0013–2022 (Wang 2023).

#### Dynamic culture in the chip

##### Perfusion in the chip

Fluid flow in the intestinal chip for cell culture can be achieved using syringe pumps, peristaltic pumps, or gravity-driven methods to meet the requirements for dynamic cell culture conditions.

##### Range of the shear stress

The range of shear stress generated by the fluid in the chip culture medium typically ranges from 0.001 dyn/cm^2^ to 10 dyn/cm^2^.

### Construction methods

#### Method for chip fabrication

The soft lithography method for preparing the intestinal chip carrier is detailed in Appendix A.

#### Method for seeding cells in the chip

The method for seeding cells in the intestine-on-a-chip is detailed in Appendix B, sections B.3.1 to B.3.3.

#### Method for culturing cells in the chip

Cell culture on the intestinal chip should comply with the provisions detailed in Appendix B, section B.3.4. The calculation method for fluid shear stress in the intestinal chip should comply with the provisions detailed in Appendix C.

## Validation of intestine-on-a-chip

### Function testing requirements

#### Intestinal villi morphology

After applying fluid shear stress to the intestinal organ chip for a certain period, dark arc-shaped, tubular, or circular villous structures should be observed, with clear boundaries.

#### Tissue barrier function

##### Immunofluorescence characterization

In the intestine-on-a-chip, the expression of one or more tight junction proteins such as ZO-1, Occludin, and Claudin-1 in epithelial cells should generally be detected.

##### TEER measurement

TEER values should typically be measured when the intestinal organ chip cells have cultured to form a complete intestinal barrier:

- When only the Caco-2 cell line is present in the intestinal organ chip, the TEER value is usually greater than 500 Ω·cm^2^.
- When co-cultured Caco-2 and HT-29-MTX cells are present in the intestinal organ chip, the TEER value is typically greater than 100 Ω·cm^2^.
- When only organoid-derived cells are present in the intestinal organ chip, the TEER value is typically greater than 200 Ω·cm^2^.

##### Intestinal barrier permeability

After the tissue barrier is fully formed in the intestinal organ chip, permeability is determined using 4 kDa dextran (Chen 2018). Generally, the apparent permeability coefficient (Papp) value should be less than 1 × 10^–6^ (1 h).

#### Specific genes expression in chip

Intestinal organ chips typically should detect the expression of marker genes ALPI (Intestinal Alkaline Phosphatase) and Villin, which are indicative of absorptive intestinal epithelial cells. When goblet cells are present in the intestinal chip, the marker gene MUC2 (Mucin 2) should also be detected (Kwon 2021).

#### Functional protein secretion in chip

When goblet cells are present in the intestinal organ chip, secretion of mucin MUC2 is typically detected.

### Validation methods for chip biofunctions

#### Observation of intestinal villi

##### Bright-field observation

During the cell culture process of the intestinal organ chip, observation should be conducted daily under an inverted phase contrast microscope to determine the appearance and morphological changes of intestinal villi.

##### SEM observation

SEM observation of intestinal villi in the intestinal organ chip should comply with the methods outlined in Appendix D.

#### Assessment of intestinal barrier

##### Immunofluorescence characterization

After the formation of the intestinal tissue barrier in the intestinal organ chip, tight junctions between cells should be visible under a microscope, with clear cell boundaries. Detection of the expression of tight junction proteins in the tissue barrier of the intestinal organ chip should comply with the methods outlined in Appendix E.

##### TEER measurement

TEER measurement is mainly based on Ohm's law and electrochemical impedance methods:

When measuring TEER based on Ohm's law, alternating current square waves are typically applied to the cells to maintain a constant small current.

When measuring TEER using the electrochemical impedance method, working electrodes and counter electrodes are typically placed on both sides of the cell layer, and the impedance of the measurement system is measured.

##### Molecular permeability detection

Molecular permeability testing of the intestinal organ chip tissue barrier should comply with the methods outlined in Appendix F, section F.3. The data processing process should comply with the methods outlined in Appendix F, sections F.4 to F.6.

#### Detection of cell marker gene expression

Detection of cell marker gene expression in the intestinal organ chip should comply with the methods outlined in Appendix G, sections G.3.1 to G.3.3. The data processing process should comply with the methods outlined in Appendix G, section G.3.4.

#### Detection of cell protein secretion

Detection of cell protein secretion in the intestinal organ chip should comply with the methods outlined in Appendix H.

## Data Availability

Not applicable.

## Appendix A

(Normative Appendix)

Chip Fabrication: Soft Lithography

- **A.1 Instruments**
  - A.1.1 Spin coater.
  - A.1.2 Hotplate.
  - A.1.3 Oven.
  - A.1.4 Ultraviolet (UV) exposure system.
  - A.1.5 Horizontal shaker.
  - A.1.6 Vacuum dryer.
  - A.1.7 Plasma cleaner.
- **A.2 Reagents**
  - A.2.1 Unless otherwise specified, all reagents used are analytical grade, and water is ultrapure water.
  - A.2.2 Photoresist.
  - A.2.3 Developer solution (negative resist developer generally contains organic solvents such as xylene and ethyl lactate; positive resist developer is usually a strong alkaline solution, such as potassium hydroxide and tetramethylammonium hydroxide).
  - A.2.4 PDMS monomer.
  - A.2.5 PDMS initiator.
  - A.2.6 Substrate: glass or silicon wafer.
  - A.2.7 Silanization agent: typically, dimethyldichlorosilane or trimethylchlorosilane.
- **A.3 Fabrication Procedures**
  - A.3.1 Substrate Pre-treatment: Drying
  - Before fabricating the chip template, ensure to place the substrate (A.2.6) in an oven (A.1.3) for at least 1 h to eliminate moisture. Set the oven temperature between 150 °C to 200 °C for this process.
  - A.3.2 Chip Template Fabrication: Coating and Pre-baking.
  - Place the substrate in the center of the spin coater (A.1.1), add photoresist (A.2.2) to the center, and adjust the rotation speed to achieve the desired thickness. Then, place the substrate on a preheated hotplate (A.1.2) for pre-baking.
  - A.3.3 Chip Template Fabrication: Exposure and Post-baking
  - Affix the pre-designed and printed chip mask onto the substrate after pre-baking, and expose it in a UV exposure system (A.1.4). The exposure intensity and time depend on the photoresist thickness. Then, post-bake the substrate on a preheated hotplate after exposure.
  - A.3.4 Chip Template Fabrication: Development and Hard Baking
  - Place the chip template, cooled to room temperature, into a suitable container. Add developer solution (A.2.3) to fully cover the substrate, and gently agitate it either on a horizontal shaker (A.1.5) or by continuous agitation with the developer solution. Stop the development process once the microstructures on the substrate are fully revealed and the edges are clear. Dry the template surface using inert gas, such as nitrogen, to remove any residual moisture. Finally, hard bake the template at temperatures ranging from 150 °C to 200 °C to ensure its stability and durability for subsequent use.
  - A.3.5 Chip Template Fabrication: Surface Passivation
  - Place the chip template, cooled to room temperature, into a vacuum dryer (A.1.6). Simultaneously, add a small amount of silanization agent (A.2.7) to the side of the template, allowing the silanization agent's gas to fully deposit on the template surface at room temperature.
  - A.3.6 Fabrication and Sealing of PDMS Chip
  - Mix PDMS monomer (A.2.4) and initiator (A.2.5) in a specified volume ratio, typically 10:1. Pour the mixture onto the chip template to achieve the desired thickness. Heat the PDMS-containing chip template in an oven at 60 °C to 100 °C for at least 30 min to cure. Once cured, peel the PDMS from the template and cut it to the appropriate size. Use a puncher to create the inlet and outlet of the chip. Subsequently, employ a plasma cleaner (A.1.7) to activate the PDMS blocks with different chamber structures and the porous membrane. Seal the activated porous membrane between two PDMS blocks in a sandwich form to complete the sealing process.

## Appendix B

(Normative Appendix)

Construction of Intestine-on-a-Chip: Cell Seeding and Culture

- **B.1 Instruments**
  - B.1.1 Ultrasonic cleaner.
  - B.1.2 Cell culture incubator.
  - B.1.3 Oven.
  - B.1.4 Optical microscope.
- **B.2 Reagents**
  - B.2.1 Unless otherwise specified, all reagents used are analytical grade, and water is ultrapure water.
  - B.2.2 75% Ethanol.
  - B.2.3 Intestinal cell culture medium: H-DMEM medium supplemented with 10% ~ 20% serum + 1% antibiotics. After preparation, store at 4 °C.
  - B.2.4 Endothelial cell culture medium: ECM medium supplemented with 10% serum + 1% endothelial cell growth supplement + 1% antibiotics. After preparation, store at 4 °C.
  - B.2.5 Intestinal organoid culture medium: Advanced DMEM/F12 medium supplemented with factors including but not limited to N-2 Supplement, Serum-Free B27, GlutaMax, and other factors as needed for the experiment.
  - B.2.6 Cell digestion solution: commonly used 0.25% Trypsin EDTA and TrypLE.
- **B.3 Procedures of Cell Seeding and Culture**
  - B.3.1 Chip Cleaning and Sterilization
  - Soak the PDMS chip in water and then place it into the ultrasonic cleaner (B.1.1) for ultrasonic cleaning. Afterwards, dry the chip in the oven (B.1.3). Subsequently, sterilize it by soaking it in 75% ethanol (B.2.2) for 10 min.
  - B.3.2 Seeding Endothelial Cells on the Chip
  - After digestion with cell digestion solution (B.2.6), suspend endothelial cells in endothelial cell culture medium to achieve a desired cell concentration. Ensure the seeding density is adequate for the cells to cover the bottom of the chamber after overnight culture. Carefully add the endothelial cell suspension from the chamber inlet to fill the channel without introducing bubbles. Allow the endothelial cells to settle naturally on the porous membrane. If endothelial cells are seeded on the underside of the porous membrane, invert the chip in the cell culture incubator at 37 °C for a specified period to allow cell adhesion. Finally, add an appropriate volume of endothelial cell culture medium (B.2.4) to fill the channel and culture the chip in the cell culture incubator.
  - B.3.3 Seeding Intestinal Epithelial Cells on the Chip
  - After seeding endothelial cells, proceed with seeding intestinal epithelial cells. Treat the cells with cell digestion solution, then prepare a cell suspension of a specified concentration using either intestinal cell culture medium (B.2.3) or intestinal organoid culture medium (B.2.5). Ensure the seeding density is sufficient for the cells to cover the channel after overnight culture. Carefully add the appropriate volume of cell suspension from the epithelial chamber inlet to fill the chamber. Incubate the chip in the incubator for 1 to 2 days to allow the cells to form a complete monolayer structure.
  - B.3.4 Dynamic Culture on Intestine-on-a-Chip
  - Place the chip containing seeded cells in the cell culture incubator (B.1.2) and connect it to the dynamic culture system. The commonly used fluid shear force in experiments ranges from 0.001 dyn/cm^2^ to 10 dyn/cm^2^. Subsequently, observe cell growth and differentiation under an optical microscope (B.1.4) and replace the culture medium as needed.

## Appendix C

(Normative Appendix)

Construction of Intestine-on-a-Chip: Calculation of Shear Force.

The calculation of fluid shear force involves the principles of fluid dynamics, mainly based on Newton's viscous fluid model. In a viscous fluid, the shear force (τ) can be calculated using Formula (1):

1 $$\tau =\mu \times du/dy$$

where:

*τ* is the shear force, measured in dyn/cm^2^

*μ* is the viscosity coefficient of the fluid, measured in (dyn/cm^2^) × s.

*du/dy* is the velocity gradient, representing the rate of velocity change of the fluid perpendicular to the direction of flow, measured in s^−1^.

## Appendix D

(Normative Appendix)

Validation of Tissue Morphology: SEM

- **D.1 Instruments**
  - D.1.1 Critical Point Dryer
  - D.1.2 Vacuum Ion Sputtering Instrument
  - D.1.3 Scanning Electron Microscope
- **D.2 Reagents**
  - D.2.1 Unless otherwise specified, all reagents used are analytical grade, and water is ultrapure water.
  - D.2.2 Anhydrous Ethanol: Prepare a solution of the required concentration with ultrapure water before use.
  - D.2.3 Phosphate Buffered Saline (PBS): Main components include Na2HPO4, KH2PO4, NaCl, and KCl, with a pH of 7.2 ~ 7.4.
  - D.2.4 Tissue Fixative: 4% paraformaldehyde solution.
  - D.2.5 0.1 M Phosphate Buffer (PB): Main components are KH2PO4 and NaOH, with a pH of 7.4.
- **D.3 Validation Procedures**
  - D.3.1 Cell Fixation
  - After a specified period of cell culture in the intestinal organ chip, remove the culture medium and wash the cells with buffer solution. Then, add an appropriate volume of tissue fixative (D.2.4) to fully immerse the cells in the intestinal organ chip. Once the cells are completely fixed, thoroughly rinse them with buffer solution in preparation for the next experiment.
  - D.3.2 Dehydration
  - Sequentially dehydrate the cells in the chip by immersing them in ethanol (D.2.2) solutions with the following volume concentrations: 30%, 50%, 70%, 80%, 90%, 95%, and two rounds of 100%, all at room temperature.
  - D.3.3 Sample Drying
  - Transfer the samples from step A.3.2 into the critical point dryer (D.1.1) and dry them according to the standard procedure for a minimum of 2 h.
  - D.3.4 Sample Coating
  - Once the cell samples are thoroughly dried, utilize the vacuum ion sputtering instrument (D.1.2) to apply a thin layer of carbon, gold, or platinum onto the surface of the samples. This coating is essential to enhance their conductivity.
  - D.3.5 SEM Observation
  - SEM (D.1.3) can be employed to examine the microvilli structure on the surface of the samples after collecting image data.

## Appendix E

(Normative Appendix)

Validation of Intestinal Barrier: Immunofluorescence

- **E.1 Instruments**
  - Confocal Laser Scanning Microscope.
- **E.2 Reagents**
  - E.2.1 Unless otherwise specified, all reagents used are analytical grade, and water is ultrapure water.
  - E.2.2 Immunofluorescence staining reagent kit.
  - E.2.3 PBS: Main components include Na2HPO4, KH2PO4, NaCl, and KCl, with a pH of 7.2 ~ 7.4.
    E.2.4 Target protein antibodies.
  - E.2.5 Tissue Fixative: 4% paraformaldehyde.
- **E.3 Validation Procedures**
  - E.3.1 Cell Fixation
  - After a specified period of cell culture in the intestinal organ chip, remove the culture medium and wash the cells with buffer solution. Then, add an appropriate volume of tissue fixative (E.2.4) to fully immerse the cells in the intestinal organ chip. Once the cells are completely fixed, thoroughly rinse them with buffer solution in preparation for the next experiment.
  - E.3.2 Immunofluorescence Staining
  - Perform immunofluorescence staining using the immunofluorescence staining reagent kit (E.2.2) and target protein antibodies (E.2.4) according to the instructions provided with the kit.
  - E.3.3 Immunofluorescence Observation
  - Utilize a confocal laser scanning microscope to observe and capture images of the stained intestinal barrier on the chip. Typically, the expression of one or more tight junction proteins (such as ZO-1, Occludin, Claudin-1, etc.) should be detected. Additionally, generate a 3D reconstructed side view based on immunofluorescence staining to measure the height of the intestinal villi.

## Appendix F

(Normative Appendix)

Detection of Molecular Permeability: Fluorescein-labeled Dextran Permeability Assay

- **F.1 Instruments**
  - Microplate reader.
- **F.2 Reagents**
  - F.2.1 Unless otherwise specified, all reagents used are analytical grade, and water is ultrapure water.
  - F.2.2 Hank's Balanced Salt Solution (HBSS) Buffer: pH 7.2 ~ 7.4.
  - F.2.3 Fluorescein-labeled Dextran Solution (FITC-Dextran): Different molecular weights ranging from 4 to 70 kDa can be selected.
- **F.3 Detection Procedures**
  - F.3.1 Preparation of Dextran Solution
  - Once the cells in the intestinal organ chip have formed a complete barrier, conduct the permeability experiment using FITC-Dextran (F.2.3). Firstly, prepare a 1 mg/ml FITC-Dextran solution in HBSS buffer (F.2.2) and set it aside. Next, wash the cells once with HBSS buffer flowing through the chip. Subsequently, replace the medium flowing through the chip with the prepared 1 mg/ml FITC-Dextran solution for perfusion incubation.
  - F.3.2 Drawing the Standard Curve
  - Prepare a standard curve of concentration versus fluorescence intensity by diluting the FITC-Dextran solution in HBSS buffer in a 96-well plate with a certain concentration gradient and determine the linear range.
  - F.3.3 Fluorescence Quantification
  - After 1 h of cell incubation, take a certain amount of effluent from the porous membrane into a 96-well plate and measure the fluorescence intensity of Dextran using a microplate reader.
- **F.4 Calculation of Apparent Permeability Coefficient**
  - Calculating the apparent permeability coefficient using Formula (2):
  - 2 $$Papp\,=\,(dQ/dt)/(A\times{c_{0}})$$

where:

- *Papp* is the apparent permeability coefficient, with units of cm/s.
- *dQ* is the total amount of Dextran permeated in a certain time, with units of mg.
- *dt* is the time of the experiment, with units of s.
- *A* is the membrane area of the chip, with units of cm^2^.
- *c*_*0*_ is the initial concentration of Dextran, with units of mg/ml.

F.5 Calculation and Analysis

Repeat the experiment, calculate the average of three times, and record it as the apparent permeability coefficient of Dextran in the chip at that molecular weight.

F.6 Result Analysis

For a processing time of 1 h, the Papp value for 4 kDa Dextran should be less than 1 × 10^–6^ for a complete intestinal barrier. The larger the molecular weight, the smaller the Papp value.

## Appendix G

(Normative Appendix)

Detection of Marker Gene: Real-time Fluorescence Quantitative PCR

- **G.1 Instruments**
  - G.1.1 PCR Instrument.
  - G.1.2 Real-time Fluorescence Quantitative PCR Instrument.
- **G.2 Reagents**
  - G.2.1 Unless otherwise specified, all reagents used are analytical grade, and water is ultrapure water.
  - G.2.2 PBS: Main components include Na2HPO4, KH2PO4, NaCl, and KCl, pH 7.2 ~ 7.4.
  - G.2.3 RNA Extraction Kit.
  - G.2.4 RNA Reverse Transcription Kit.
  - G.2.5 Fluorescence Quantitative PCR Amplification Kit.
  - G.2.6 GAPDH and target gene qPCR primers.
- **G.3 Detection Procedures**
  - G.3.1 RNA Extraction
  - Wash the intestinal cells to be tested in the chip with buffer. Use the RNA extraction kit (G.2.3) to extract total RNA from the cells in the chip, following the instructions of the kit.
  - G.3.2 cDNA Preparation
  - Take the RNA extracted in D.3.1 and perform reverse transcription using the Polymerase Chain Reaction (PCR) instrument (G.1.1) with the RNA reverse transcription kit (G.2.4), following the instructions of the kit and instrument.
  - G.3.3 Determination of Gene Expression
  - Take the RNA reverse transcription products obtained in step G.3.2, and employ the real-time fluorescence quantitative PCR instrument (G.1.2). Utilize the fluorescence quantitative PCR amplification kit (G.2.5) along with the relevant primers (G.2.6) to conduct real-time fluorescence quantitative detection, following the kit instructions. Refer to the detection curve to determine the Cq value, thereby obtaining the expression values Cq (GADPH) and Cq (X) of the target gene.
  - G.3.4 Analysis of Target Gene Expression
  - Using GAPDH as the internal reference gene, calculate the relative expression level of the target gene using the 2^−ΔΔCt^ method.

## Appendix H

(Normative Appendix)

Detection of Cell Mucin: Mucin Staining

- **H.1 Instruments**
  - Optical microscope.
- **H.2 Reagents**
  - H.2.1 Unless otherwise specified, all reagents used are analytical grade, and water is ultrapure water.
  - H.2.2 PBS: pH 7.2 ~ 7.4.
  - H.2.3 4% Formaldehyde Solution.
  - H.2.4 Mucin Staining Method Reagent Kit.
- **H.3 Detection Procedures**
  - H.3.1 Sample Preparation
  - After a specific period of cell culture in the intestinal chip, remove the culture medium, wash the cells with buffer, and then immerse the cells completely in an appropriate amount of tissue fixative solution (H.2.4). Once the cells are fully fixed, wash them with buffer in preparation for the subsequent step of the experiment.
  - H.3.2 Mucin Staining
  - Perform mucin staining on the cells in the intestinal chip using the Mucin Staining Method Reagent Kit (H.2.4), following the instructions provided with the kit.
  - H.3.3 Microscopic Observation
  - Observe and photograph the stained cells using an optical microscope.
  - H.3.4 Analysis of Mucin
  - Based on the staining results obtained from the Mucin Staining Method Reagent Kit, assess whether mucin expression is detected in the intestinal chip.

## Abbreviations

AIDS: Acquired Immune Deficiency Syndrome
ATCC: American Type Culture Collection
HBV: Hepatitis B Virus
HCV: Hepatitis C Virus
HIV: Human Immunodeficiency Virus
Papp: Apparent Permeability Coefficient
PC: Polycarbonate
PCR: Polymerase Chain Reaction
PDMS: Polydimethylsiloxane
PET: Polyethylene Terephthalate
PMMA: Polymethyl Methacrylate
PS: Polystyrene
RNA: Ribonucleic Acid
SEM: Scanning Electron Microscope
TEER: Transepithelial Electrical Resistance
3D: Three Dimensional
